# Supplementary material for: Learning speed and detection sensitivity controlled by distinct cortico-fugal neurons in visual cortex
Source: eLife. 2020 Dec 7;9:e59247. doi: 10.7554/eLife.59247 (PMC7748414; doi:10.7554/eLife.59247)
Supplement: Transparent reporting form [file elife-59247-transrepform.docx]

***eLife’s* transparent reporting form**

We encourage authors to provide detailed information *within their submission* to facilitate the interpretation and replication of experiments. Authors can upload supporting documentation to indicate the use of appropriate reporting guidelines for health-related research (see EQUATOR Network), life science research (see the BioSharing Information Resource), or the ARRIVE guidelines for reporting work involving animal research. Where applicable, authors should refer to any relevant reporting standards documents in this form.

If you have any questions, please consult our Journal Policies and/or contact us: editorial@elifesciences.org.

**Sample-size estimation**

- You should state whether an appropriate sample size was computed when the study was being designed
- You should state the statistical method of sample size computation and any required assumptions
- If no explicit power analysis was used, you should describe how you decided what sample (replicate) size (number) to use

Please outline where this information can be found within the submission (e.g., sections or figure legends), or explain why this information doesn’t apply to your submission:

Sample sizes were not statistically predetermined but were based on previous experiments conducted by us and other labs, that used similar experimental techniques and behavioral paradigms. The actual sample size for each experiment is listed in the main text and/or the corresponding figure legend.

**Replicates**

- You should report how often each experiment was performed
- You should include a definition of biological versus technical replication
- The data obtained should be provided and sufficient information should be provided to indicate the number of independent biological and/or technical replicates
- If you encountered any outliers, you should describe how these were handled
- Criteria for exclusion/inclusion of data should be clearly stated
- High-throughput sequence data should be uploaded before submission, with a private link for reviewers provided (these are available from both GEO and ArrayExpress)

Please outline where this information can be found within the submission (e.g., sections or figure legends), or explain why this information doesn’t apply to your submission:

Individual mice were considered as independent biological replicates. The number of samples is listed together with the measurements in the manuscript. All of the observations have been successfully reproduced in multiple cohorts for animals.

To assess the impact of VC on contrast sensitivity through the CT pathway, mice that did not exhibit a cortical contribution of VC on contrast sensitivity before the actual CT lesion were excluded as an indication that these animals already entered a training-independent stage in which the VC lost its contribution to the task performance and thus did not permit the assessment of the selective role of the CT pathway.

**Statistical reporting**

- Statistical analysis methods should be described and justified
- Raw data should be presented in figures whenever informative to do so (typically when N per group is less than 10)
- For each experiment, you should identify the statistical tests used, exact values of N, definitions of center, methods of multiple test correction, and dispersion and precision measures (e.g., mean, median, SD, SEM, confidence intervals; and, for the major substantive results, a measure of effect size (e.g., Pearson's r, Cohen's d)
- Report exact p-values wherever possible alongside the summary statistics and 95% confidence intervals. These should be reported for all key questions and not only when the p-value is less than 0.05.

Please outline where this information can be found within the submission (e.g., sections or figure legends), or explain why this information doesn’t apply to your submission:

All statistical tests used for the data analysis are listed together with the corresponding data, the exact values of N and the precision measures throughout the manuscript. The respective p-values or p-value thresholds that were used to denote statistical significance are also listed in conjunction with the corresponding data throughout the main text manuscript. The criteria to determine the statistical significance are summarized in the Materials and Methods section of the manuscript.

(For large datasets, or papers with a very large number of statistical tests, you may upload a single table file with tests, Ns, etc., with reference to sections in the manuscript.)

**Group allocation**

- Indicate how samples were allocated into experimental groups (in the case of clinical studies, please specify allocation to treatment method); if randomization was used, please also state if restricted randomization was applied
- Indicate if masking was used during group allocation, data collection and/or data analysis

Please outline where this information can be found within the submission (e.g., sections or figure legends), or explain why this information doesn’t apply to your submission:

Mice were randomly assigned to experimental groups. In case multiple mice from a single litter were used, the animals were randomly spread across groups in order to avoid a bias due to litter effects. The experimenters were blind to the experimental groups during the behavioral training and task performance assessment.

**Additional data files (“source data”)**

- We encourage you to upload relevant additional data files, such as numerical data that are represented as a graph in a figure, or as a summary table
- Where provided, these should be in the most useful format, and they can be uploaded as “Source data” files linked to a main figure or table
- Include model definition files including the full list of parameters used
- Include code used for data analysis (e.g., R, MatLab)
- Avoid stating that data files are “available upon request”

Please indicate the figures or tables for which source data files have been provided:

Numerical data for graphs represented in figures 1-6, figure 1-figure supplement 2,3,5,6, figure 2-figure supplement 1, are provided as source data files.
